# Simultaneous bilateral total knee and ankle arthroplasty as a single surgical procedure

**DOI:** 10.1186/1471-2474-12-233

**Published:** 2011-10-13

**Authors:** Geert Pagenstert, Beat Hintermann

**Affiliations:** 1 Department of Orthopaedic Surgery, University Clinics of Basel, Spitalstr. 21, CH-4031 Basel, Switzerland; 2 Department of Orthopaedic Surgery, Kantonsspital Liestal, Rheinstr. 26, CH-4410 Liestal, Switzerland

## Abstract

**Background:**

Simultaneous osteoarthritis (OA) of the ankle joint complicates primary total knee arthroplasty (TKA). In such cases, rehabilitation of TKA is limited by debilitating ankle pain, but varus or valgus ankle arthritis may even compromise placement of knee prosthetic components.

**Case presentation:**

We present a patient with simultaneous bilateral valgus and patellofemoral OA of the knees and bilateral varus OA of the ankle joints that equally contributed to overall disability. This 63 years old, motivated and otherwise healthy patient was treated by simultaneous bilateral total knee and ankle arthroplasty (quadruple total joint arthroplasty, TJA) during the same anesthesia. Two years outcome showed excellent alignment and function of all four replaced joints. Postoperative time for rehabilitation, back to work (6th week) and hospital stay (12 days) of this special patient was markedly reduced compared to the usual course of separate TJA.

**Conclusions:**

Simultaneous quadruple TJA in equally disabling OA of bilateral deformed knees and ankles resulted in a better functional outcome and faster recovery compared to the average reported results after TKA and TAA in literature. However, careful preoperative planning, extensive patient education, and two complete surgical teams were considered essential for successful performance. To the best of our knowledge this is the first case report in literature about quadruple major total joint arthroplasty implanted during the same anesthesia in the same patient.

## Background

Total joint arthroplasty (TJA) of the knee (TKA) and ankle (TAA) are well established procedures demonstrated to provide good long-term results in terms of reduced pain and increased function [1,2]. In the presence of bilateral osteoarthritis (OA) of the knees and ankles contributing equally to cumulative gait inability in the same patient, surgical treatment of all four joints may be indicated. Surgery can be accomplished in a staged fashion or as a simultaneous procedure under one anesthesia to shorten disability and rehabilitation time that would accumulate with sequential TJA.

To our knowledge, there have been no special reports on simultaneous bilateral TAA and outcome was usually incorporated in overall reports on TAA [2]. In contrast, bilateral TKA have been extensively discussed in literature [3-8]. Simultaneous bilateral TKA has been shown to be associated with higher complication rates than staged bilateral or unilateral TKA [9]. However, reduction in costs and rehabilitation time and improvement of surgical technique [5,10] with critical patient selection [4,8] has led to significant reduction of complications and further recommendation of the simultaneous procedure [4,5,8]. Current studies found even better functional outcome and patient survival in simultaneous bilateral TKA compared to unilateral TKA [11,12].

We present the unusual case of a patient who had bilateral valgus and patellofemoral OA of the knees and bilateral varus OA of the ankle joints that equally contributed to overall disability. This highly motivated and otherwise healthy patient was treated by simultaneous bilateral total knee and ankle arthroplasty in the same anesthesia. Our patient was informed that data concerning the case would be submitted for publication, and he consented.

## Case Presentation

A sixty-three-year-old executive consultant manager presented to us with known osteoarthritis (OA) in both ankles and knees since years and incremental debilitating pain since months. Valgus-extension contracture in both knees and varus-equines contracture in both ankles have reduced his maximum walking distance to about 1 km in 20 min despite crutches, bilateral custom-made lower leg orthotics and daily pain medication. He was increasingly restricted to work at his home office; needed two crutches to rise from a chair, because of bilateral knee pain; descended stairs backwards, because of bilateral knee and ankle pain. His history revealed an otherwise healthy patient, no medications other than for pain, height 178 cm, weight 92 kg, with status post right knee arthroscopic joint lavage and medial meniscectomy about 12 years ago, and repetitive ankle sprains since youth which have been managed non-surgically. Physical therapy and multiple intraarticular injections with steroids and anesthetics have lost its long-term benefit at each of the four joints. To continue his own business he was seeking surgical help.

**Figure 1:**
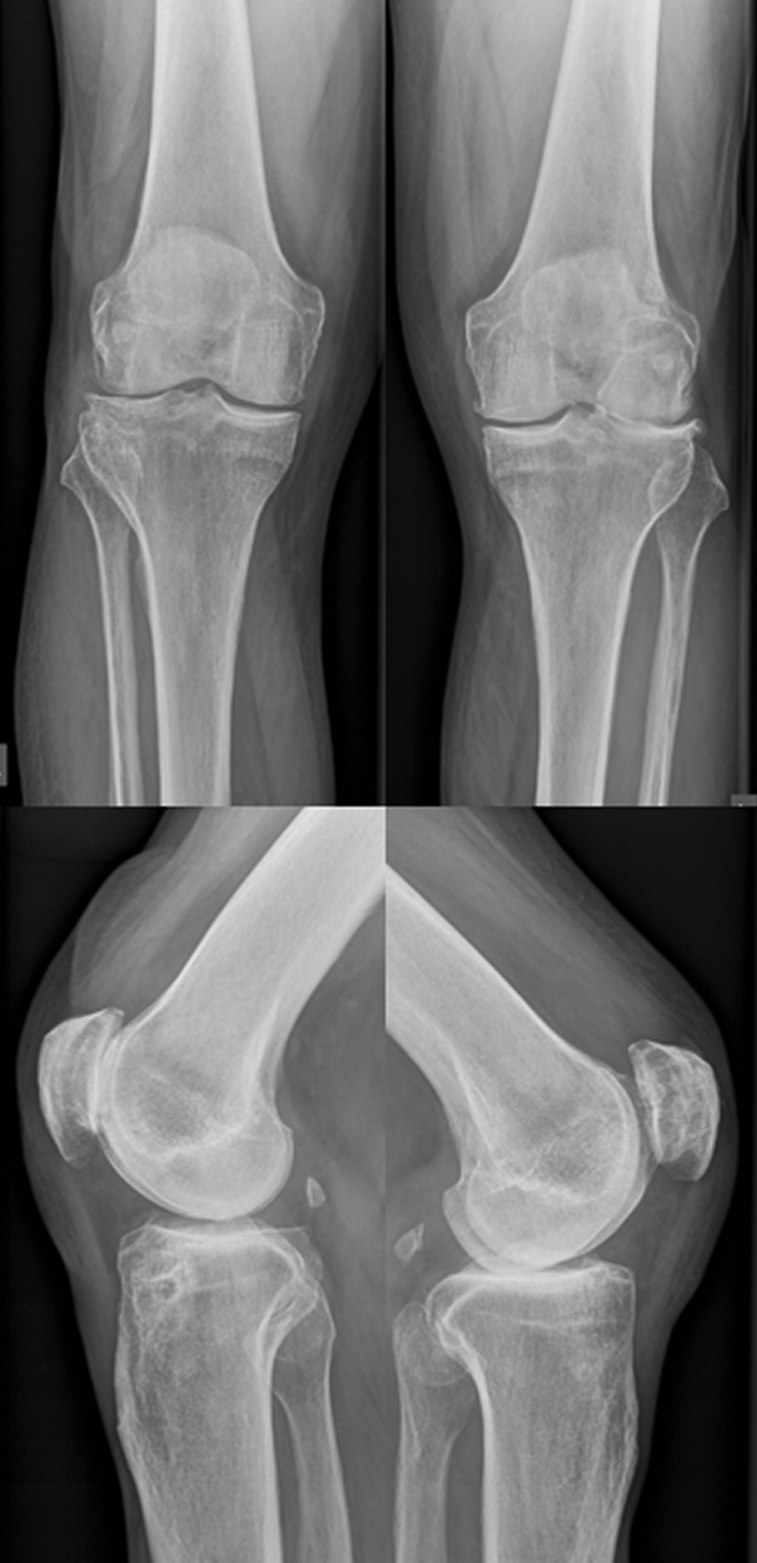
**Preoperative radiographs of the knees**. Tibiofemoral accentuated gonarthritis at the left side.

**Figure 2:**
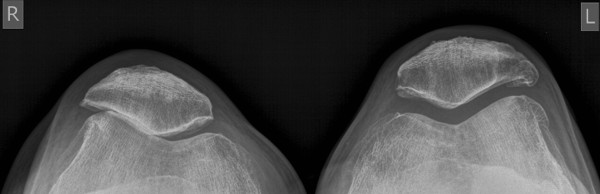
**Preoperative skyline view of the knees**. Patellofemoral accentuated gonarthritis at the right side.

On physical examination at time presented to us, both knees showed mild valgus alignment but showed an intercondylar distance of 2 cm in standing position with both feet in contact because of severe varus alignment of the foot and ankle. Both knees had intraarticular effusion and diffuse joint space tenderness. On stress testing both knees were stabile, the right knee showed maximum tenderness VAS 6-8 (visual analogue scale: minimum 0, maximum pain 10 points) at the patellofemoral joint and the left knee VAS 5-7 at the lateral tibiofemoral joint. Range of motion (ROM) was decreased at the right knee to flexion contracture of 20°, deep flexion of 120°, and at the left knee to flexion contracture of 15°, deep flexion of 110°. Standing plain radiographs showed a patellofemoral accentuated gonarthritis on the right- and a lateral tibiofemoral accentuated gonarthritis on the left side (Figure 1, 2).

**Figure 3:**
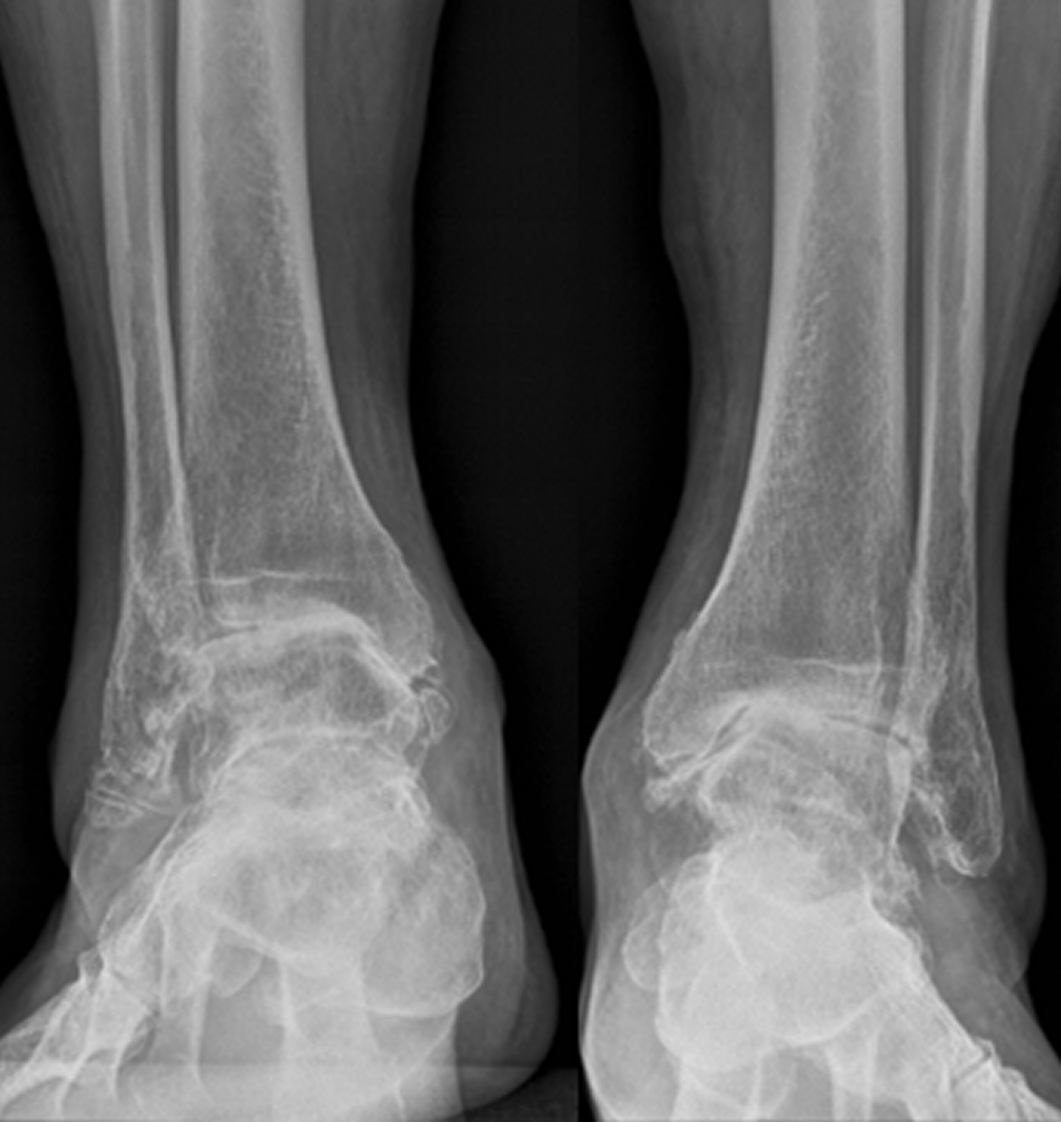
**Preoperative anterior radiographs of the ankles**. Advanced ankle osteoarthritis with severe varus malalignment on both sides

**Figure 4:**
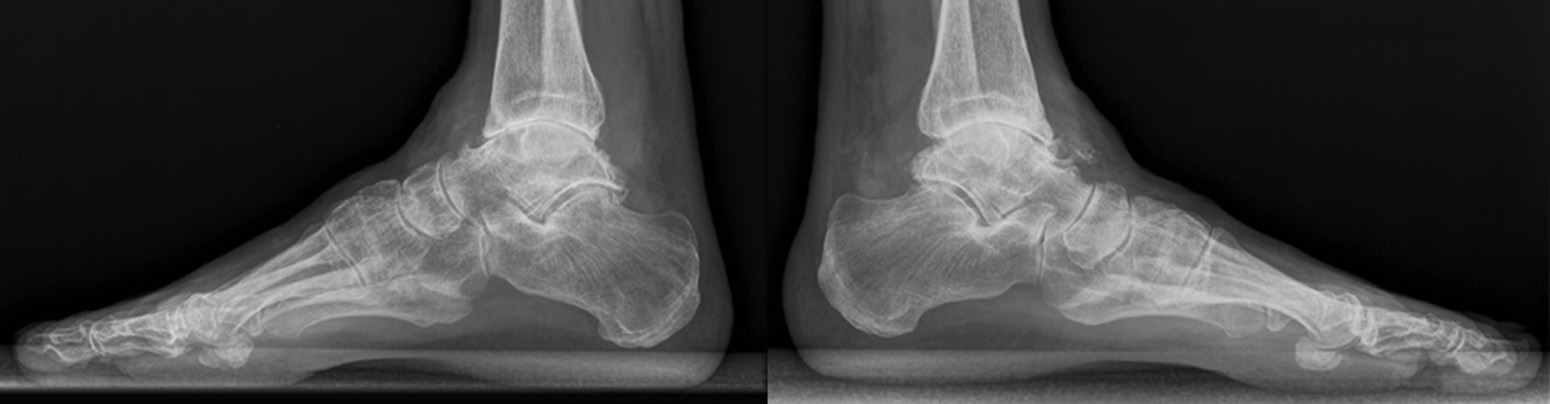
**Preoperative lateral views of the ankles**. Concentric end-stage ankle osteoarthritis on both sides.

Both feet showed marked varus alignment of the heel, rather rigid than unstable joints with diffuse ankle joint pain VAS 8-9. The right ankle presented an impaired active pronation. ROM was decreased at the right ankle to equines position in dorsiflexion of 20°, full plantarflexion of 40°, and at the left ankle to dorsiflexion of 0°, full plantarflexion of 35°. Pro- and supination of both feet were decreased to about 50% compared to healthy population. Standing plain radiographs showed advanced ankle joint osteoarthritis with varus alignment (Figure 3, 4).

**Figure 5:**
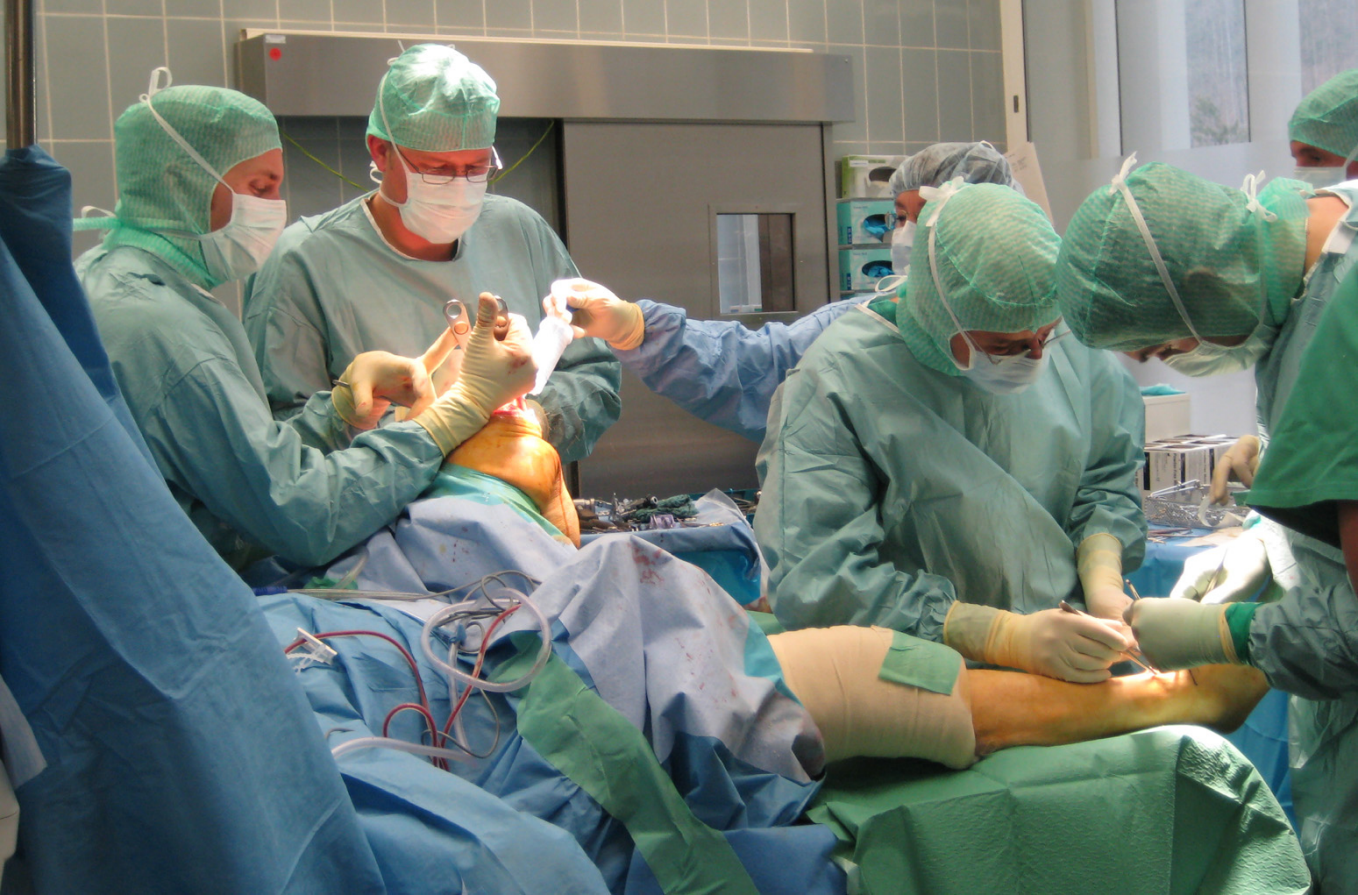
**Surgical setup during quadruple total joint arthroplasty**. Two surgical teams (surgeon, assistant, nurse) worked simultaneously on each leg. The picture shows the surgical setup at the time when the right TKA has been completed and one surgeon is implanting the TAA, while on the left leg the TAA has been completed and the other surgeon is implanting the TKA.

**Figure 6:**
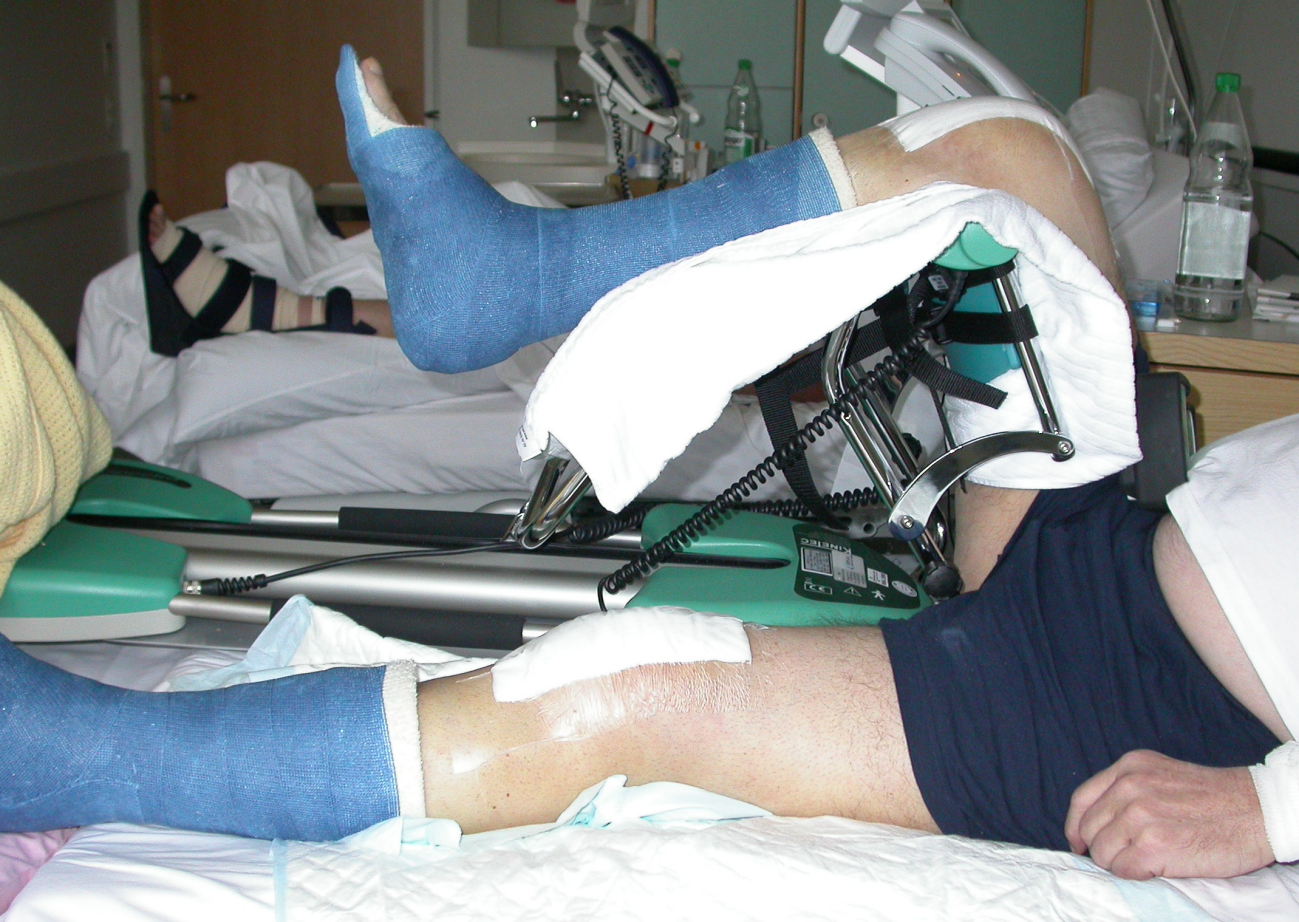
**Clinical situation on third postoperative day**. Knee flexed up to 90° with both ankles protected in rigid scotch-casts allowing full weight bearing.

The patient was educated in detail about non-surgical-, surgical joint salvage-, ankle fusion and TJA options and decided to have bilateral TKA and TAA as one surgical procedure. Surgery was performed with two surgical teams (surgeon [both authors], assistant, nurse, respectively) working at each leg, starting with the ankle on the left and the knee on the right side using a tourniquet on both sides (Figure 5). Cemented Triathlon cruciate retaining tri-compartmental TKA (Stryker, Kalamazoo, MI) via medial parapatellar approach and cementless Hintegra TAA (Integra, Plainsboro, NJ) were implanted in standard fashion, described elsewhere [13,14]. In addition, at the knees suction-drainage of the femoral canal before fluted alignment-rod insertion to decrease emboli, as well as lateral ligament repair on both ankles and a peroneus longus to brevis tendon transfer at the right ankle have been done for lateral ankle instability and right-sided peroneal dysbalance. Ankle instability became obvious after ankle osteophytes have been removed. Total surgical time from first incision to last closure was 2 hours and 29 minutes plus a total of 16 minutes for applying compression dressing at all joints. Intraoperatively, arterial pressure, pulmonary arterial pressure and pulmonary vascular resistance were observed by the anesthesiologists to abort the following TJA in case of sustained pathologic values. Perioperative intravenous cefuroxime was administered tree times per 24 hours as antibiotic prophylaxis and the patient was set on subcutaneous enoxaparin 40 mg every day as thrombembolic prophylaxis. Postoperative monitoring was continued at the intensive care unit (ICU) for 24 hours. No intra- or perioperative complication occurred. The drains in knees and ankles delivered in total 1270 ml during 48 hours after surgery. Two units of blood where given at the second postoperative day and the patient was overlapped to phenprocoumon (warfarin like drug) aiming for an international normalized ratio (INR) between 2 and 3. Physical therapy was started at the first postoperative day with knee passive range of motion. At the second postoperative day wound dressings were exchanged and bilateral non-removable lower leg scotch-casts were adjusted to both ankles to allow full weight bearing with crutches (Figure 6). Normal pain management with ibubrufen 600 mg and paracetamol 500 mg each three times a day, was enough to keep his pain between VAS 2 and 6. At the eights postoperative day the patient complained about mild dizziness with walking. A decreased hemoglobin concentration of 7.8 mmol/l was the only pathological finding and the patient recovered immediately after administration of additional two units of blood. The patient was discharged at postoperative day twelve (usually, discharge would be day 7 for TAA and 7 to 10 for TKA as unilateral procedure) and asked to continue using phenprocoumon and knee/gait physical therapy with crutches up until reappointment after two months. The patient went back to sedentary work the following week and resumed long distance aircraft travelling for a business meeting with casts and crutches (similar to preoperative condition) in the 6^th ^week after surgery.

Lower leg casts have been exchanged to control wound healing and to remove sutures of both ankles by the family physician 2 weeks after surgery. Final cast removal has been done 2 months after surgery. All wounds healed uneventfully; all joints were stabile without effusion. Range of motion was documented for knees extension/flexion 0-0-120°, and ankles dorsi-/plantarflexion 10-0-30°. Plain standing radiographs showed correct alignment and no signs of loosening of any implant. Compressive stockings were prescribed, phenprocoumon continued to a total of 5 months postoperative, physical therapy extended to the ankles and reappointment was set at one year after surgery. The patient went back to full work capacity 3 months after surgery, has discontinued all pain medications, and was able (for the first time since years) to finish 18 holes golf course by feet (in 5 hours walking on uneven ground, using regular hiking boots) 6 months after surgery.

**Figure 7:**
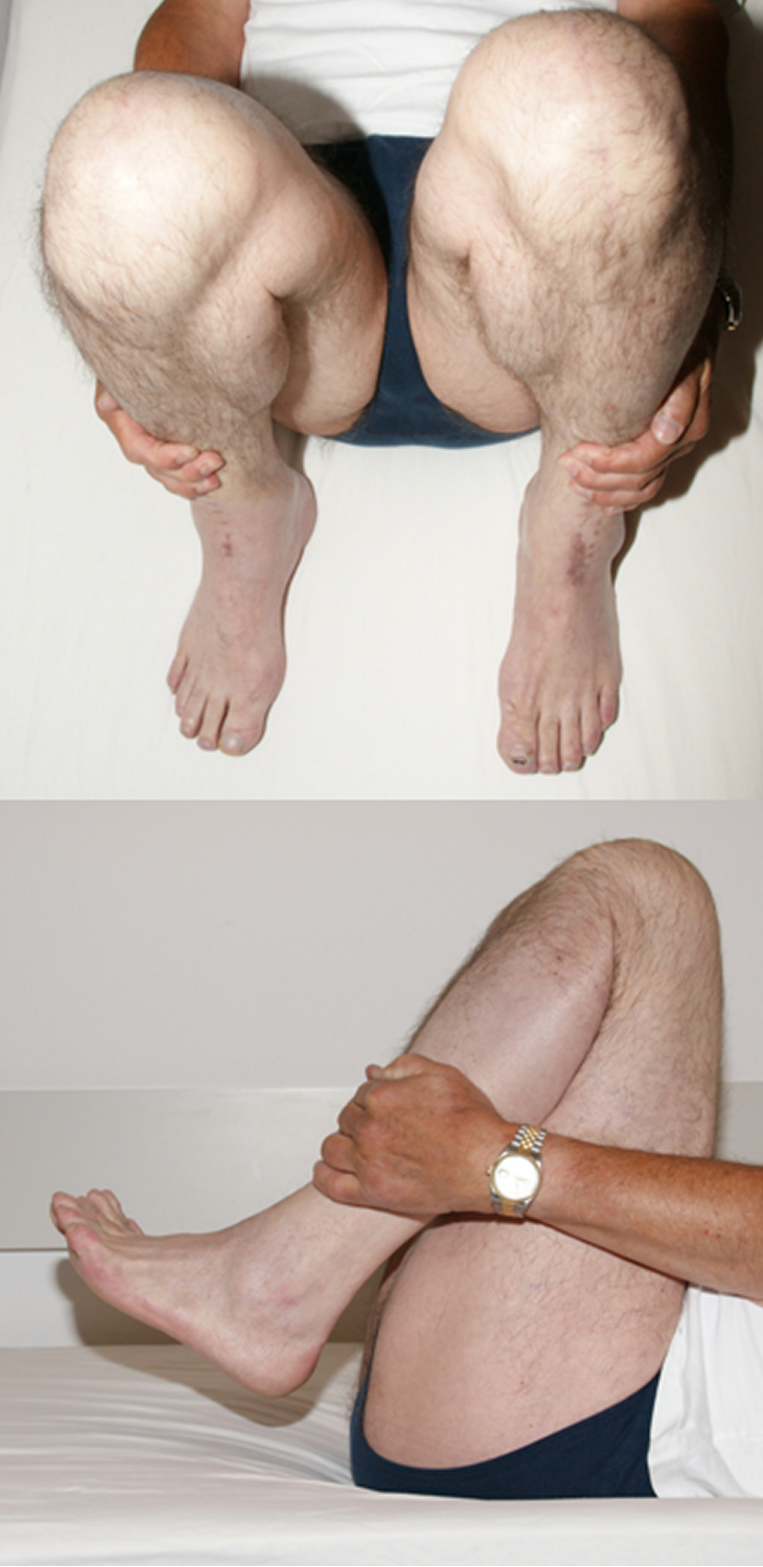
**Clinical situation 14 months after surgery**. High flexion of the knees up to 150° with heels to buttocks was possible.

At last follow-up, 25 months after surgery the patient presented with painless, stabile knees which did not limit his work or recreational activities and symmetric knee extension/flexion 5-0-150° (Figure 7). Most time during the days the patient was not aware of having TKA ("forgotten TKA"). Both ankles were clinically stabile; the left ankle was painless with dorsi-/plantarflexion ROM 15-0-40° and the right ankle mild painful (only after more than 30 min walking, VAS 3-4), ROM 15-0-30°. He was able to wear regular business shoes at work but favored regular hiking boots during recreation activities because of right-sided feeling of ankle instability walking on uneven ground. To class this individual outcome, the currently established American-Orthopaedic-Foot-and-Ankle-Society hindfoot score [15] was calculated (AOFAS-score: min. 0, max. 100 points: 40 points for pain, 50 for function, and 10 for alignment). The score increased at the right ankle from preoperative to last follow-up from 15 to 81 points, left ankle from 15 to 97 points.

**Figure 8:**
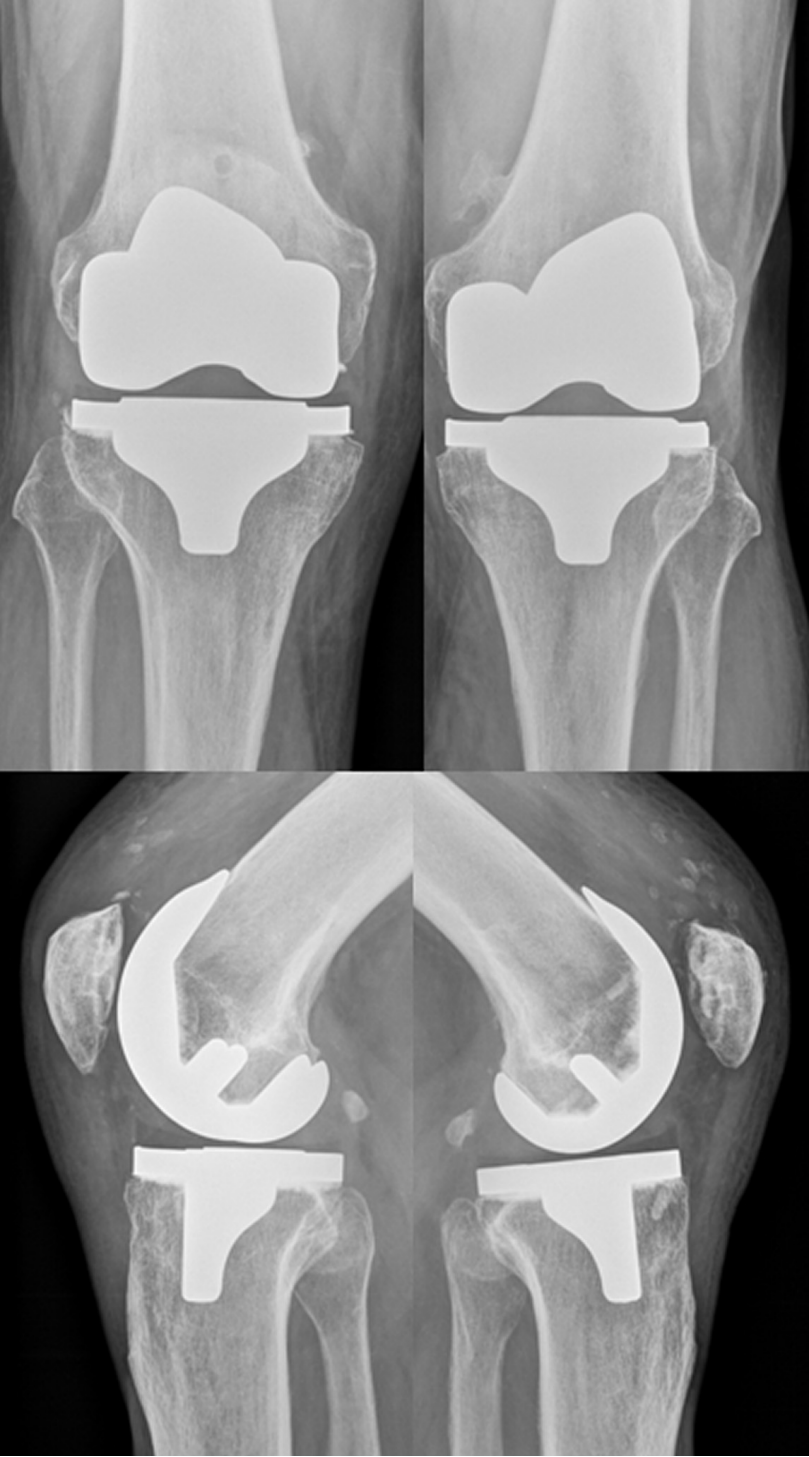
**Radiographs ap and lateral of the knees 25 months after surgery**. The picture shows correctly implanted bilateral TKAs.

**Figure 9:**
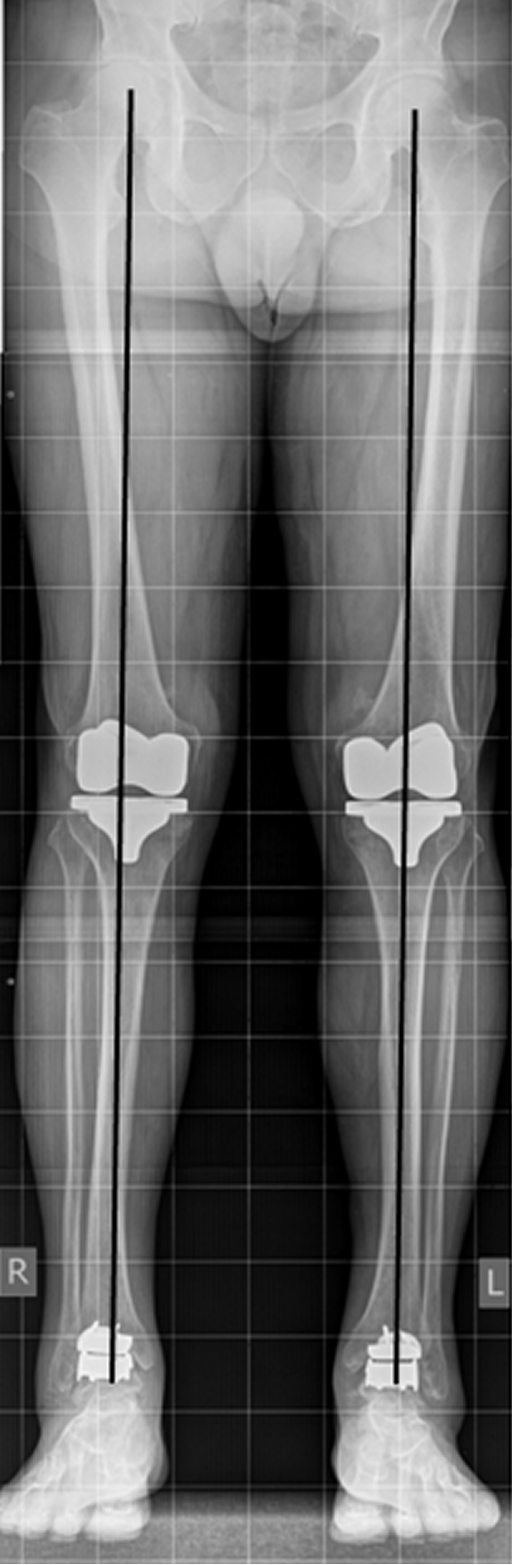
**Orthoradiogram: Orthoradiogram showing correct alignment of both TKAs and TAAs with weight bearing line running through each joint center and no signs of implant loosening**.

**Figure 10:**
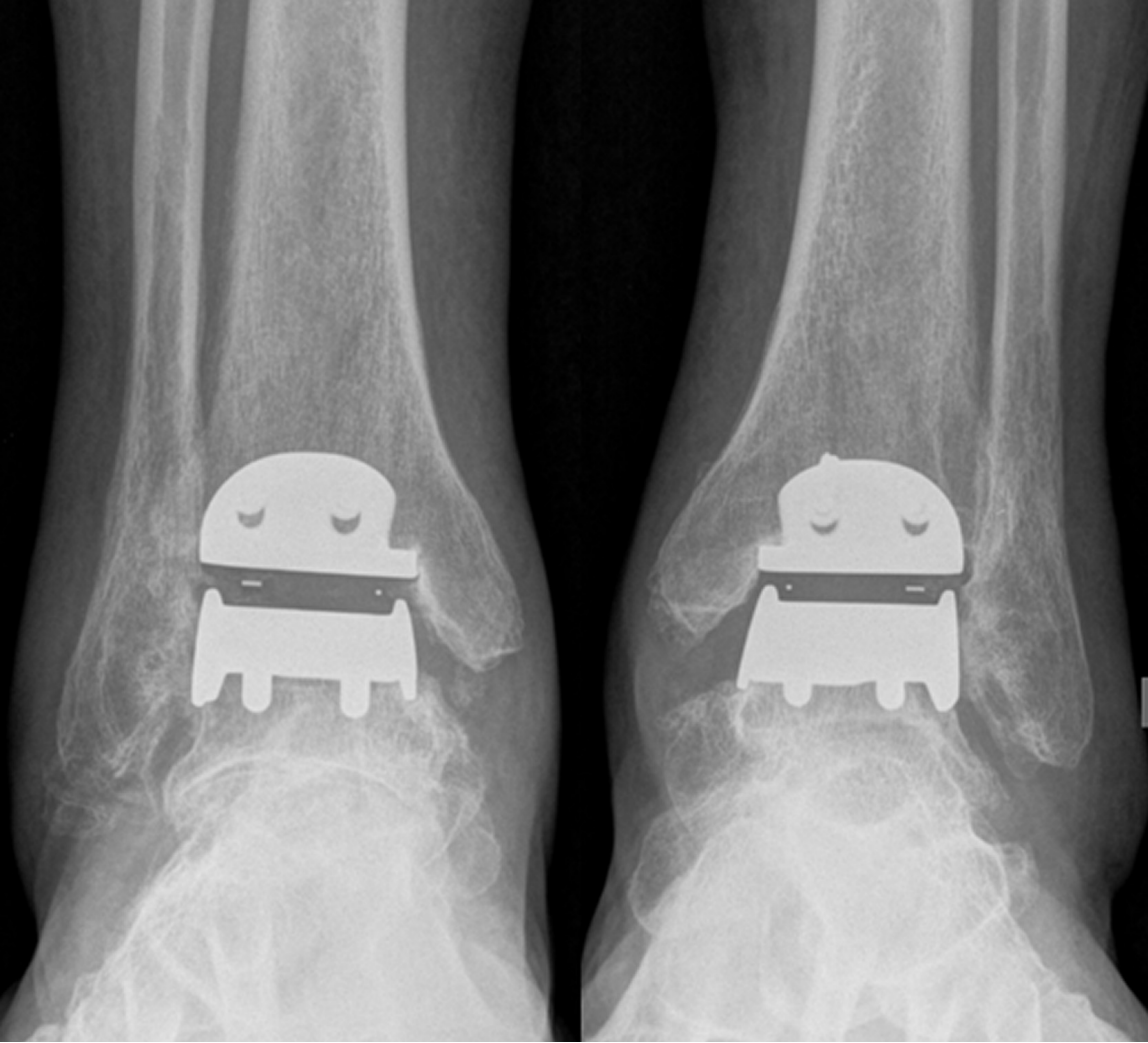
**Anterior radiographs of both ankles 25 months after TAA**. The picture shows bilateral TAAs with corrected varus alignment.

**Figure 11:**
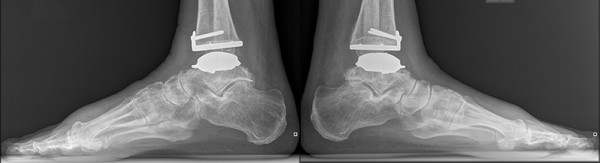
**Lateral radiographs of both ankles 25 months after TAA**. TAA centered correctly under the tibial axis.

On standing radiographs at 25 months after surgery (Figure 8, 9, 10, 11), stabile implants with correct alignment have been documented without subsidence compared to the two months control. The patient was absolute enthusiastic about being able to walk more than five hours (he did not try harder) and would recommend this treatment to a close friend. At that time the patient was sixty-five-year-old and did not think about retirement and was looking forward continuing his own business in future.

This special case illustrates that simultaneous bilateral TKA and TAA as a quadruple procedure reduced disability without major complications. To our knowledge, there have been no previous reports of a simultaneous quadruple major TJA in the literature.

In contrast, simultaneous TJA of two joints (simultaneous knees, simultaneous hips, simultaneous ipsilateral hip and knee during one anesthesia) versus staged bilateral TJA during different anesthesias have been studied extensively. Two TJA during one anesthesia was found to offer a multitude of advantages including patient convenience, shorter disability and recovery periods, and reduced costs for patients and institutions [3,6,8]. Our case report supports these findings. Currently knee flexion can be expected to be on average between 110 and 125° with excellent results in 70% of cases [1,9]. Our patient was able to flex to 150° with at most times "forgotten" TKAs. For the ankles outcome is less predictable. According to current reports, average outcomes have been reported as: combined dorsi-plantarflexion ROM of about 22.7°, AOFAS-score of 78.2 points, decreased but persistent mild pain, and an excellent result in only about 38% of cases [2]. Our patient had left ankle: no pain, ROM 55°, AOFAS-score 97; right ankle: mild intermittent pain, ROM 45°, AOFAS-score 81 points. This superior outcome after quadruple TJA may be caused in part by positive interaction of simultaneous replacements of all disabled joints as it has been described elsewhere [3,16,17]. However, in our special patient, we have to quote the strong commitment to gain independent walking ability and get back to work.

Simultaneous bilateral TKA has been found significant more often associated with severe perioperative complications as myocardial infarction, pulmonary emboli or even mortality when compared to staged bilateral TKA in patients with co-morbidities and age over 70 years [4,8]. However, in healthy and younger candidates, like our patient, no elevated risk has been detected [8]. The accumulated surgical trauma and blood loss of the simultaneous approach during one anesthesia was thought to exceed the capacity mounting compensatory physiological responses in morbid and old aged patients.

It has been shown that medical complications of simultaneous bilateral TKA can be reduced by meticulous planning together with the anesthesiologist [5]. Adjusted tourniquet use [5] and operation-time less than 2.5 hours per TKA [18] was reported to significantly reduce blood loss, infection, and revision surgery [5,18]. Total operating time of our patient was 2 h 29 min and 1270 ml blood loss of all four TJA during first 48 h postoperatively. This was comparable to an average operation-time of 2 h 16 min [5] and 1299 ml blood loss for two TKA per 48 h [8]. However, pulmonary embolism remains the leading cause of perioperative mortality and morbidity in patients undergoing TKA [5]. During TKA we used overdrilling of the femoral entry hole and irrigation of the femoral canal before insertion of the fluted intramedullary alignment rod. This technical details have been shown in literature to significantly reduce production of embolic loads [10]. In addition, we used intraoperative catheter monitoring of arterial and pulmonary arterial pressures for early detection of relevant disturbances to abort transition to next TJA, if necessary [5]. Finally, administration of oral warfarin for 3 months after TKA have been found to reduce effectively postoperative and delayed thrombembolic events [19]. Our patient received phenprocoumon for 5 months postoperative to decrease risk of thromembolic complications during rehabilitation.

OA of more than two major weight bearing joints in the same patient, i.e. bilateral hip and knee OA, have been always managed sequentially in the past [16]. However, the non-operated hip or knee joint was left deformed or restricted in mobility. It has been reported that such decreased joint function negatively influences joint posture, locomotion and overall rehabilitation of the replaced joints, frequently causing recurrence of flexion contracture at the replaced hip or knee joints [3,10,15]. Therefore quadruple TJA staged to ipsilateral simultaneous total hip and knee with a short interval of two weeks between both sides have been recommended [20]. This relatively short period seemed to be a reasonable compromise between the two aims of multiple TJA: long enough time-interval between TJA to allow regeneration from surgical trauma to reduce medical morbidity and mortality down to rates of single TJA; but short enough to prevent limited rehabilitation capacity and consecutive adhesions caused by the neglected arthritic joints. Recently, Farquhar and Snyder-Mackler showed in a prospective comparative study that the functional condition of the non-operated knee was the primary predictor of the functional outcome after unilateral TKA [17]. However, there is no literature to support that both TJA during one anesthesia has advantages over a short two weeks interval between both TJA [3,10,15]. Based on these findings it seems to be crucial to treat al disabled major joints of one patient within a short time interval to increase functional overall outcome but control fatal medical complications. When we would consider sequential TJA for combined knee and ankle OA in our patient, a correct TKA done without treatment of the ankle would have increased the varus deformity of the ankle and might have hindered full weight bearing of the limb. On the other hand a primary TAA would have experienced a change in alignment during secondary TKA. Therefore, primary TKA with consecutive TAA during one anesthesia seems to be most advantageous in such setting.

The recommended sequence during multiple TJA at the same limb was proposed to implant "from proximal to distal" to control alignment. For example, a deformed arthritic hip may confuse proper placement of femoral knee implant if TKA is done before THA. On the other hand, even a correct aligned primary TKA may experience a critical change in alignment during secondary THA [20]. Although we could not find a report about TKA and TAA during one anesthesia in the literature, we believe that severely deformed ankle OA may have a similar influence on TKA as described for hip OA. However, in our case there were no huge deformities at the knee or tibial shaft, therefore one surgical team worked at the ankle and the other simultaneously at the knee to decrease disturbance between teams, decrease instruments on the operative tables, and increase speed of overall surgical time. Therefore at the right leg the TAA was done before TKA. In addition, postoperative limb alignment was equal to the left side because alignment was adjusted to the tibial shaft in TAA and TKA. Therefore the rule to replace major joints from proximal to distal may be less important in combined TKA and TAA.

## Conclusions

Replacements of deformed and disabled arthritic knee and ankle joints within a short period of time have beneficial influences on each other and improve overall rehabilitation and patients' outcome. However, primary decision for simultaneous TJA should be based on the medical condition of the patient to tolerate multiple surgical trauma.

## Abbreviations

OA: osteoarthritis; TKA: total knee arthroplasty; TJA: total joint arthroplasty; TAA: total ankle arthroplasty; VAS: visual analogue scale; ROM: range of motion; ICU: intensive care unite; INR: international normalized ratio; AOFAS-score: score of the American Orthopaedic Foot and Ankle Society

## Competing interests

BH was involved in the development of the ankle prosthesis used in the published article and receives institutional research royalties from INTEGRA Company. GP has no competing interests in relation to the published material.

## Authors' contributions

Both authors did the surgical procedures and the follow-up examinations. GP wrote the manuscript and BH did corrections. Both authors have read and approved the revised manuscript.

## Pre-publication history

The pre-publication history for this paper can be accessed here:

http://www.biomedcentral.com/1471-2474/12/233/prepub
